# The impact of foreign accent on irony interpretation

**DOI:** 10.1371/journal.pone.0200939

**Published:** 2018-08-08

**Authors:** Sendy Caffarra, Elissa Michell, Clara D. Martin

**Affiliations:** 1 BCBL, Basque Center on Cognition, Brain and Language, Paseo Mikeletegi 69, Donostia, Spain; 2 Ikerbasque, Basque Foundation for Science, Maria Diaz de Haro 3, Bilbao, Spain; Fordham University, UNITED STATES

## Abstract

In modern multi-cultural societies, conversations between foreign speakers and native listeners have become very common. These exchanges often include the use of figurative language. The present study examines, for the first time, whether native listeners’ non-literal interpretation of discourse is influenced by indexical cues such as speaker accent. Native listeners were presented with ironic and literal Spanish stories uttered in a native or foreign accent (Spanish and British English accents, respectively). Two types of irony were considered: ironic criticism (frequently used) and ironic praise (less frequently used). Participants were asked to rate stories on their level of irony. Results showed an impact of foreign accent on natives’ non-literal interpretation. The effect was evident in the less frequent ironic constructions (ironic praise), with foreign accented utterances considered less ironic than native accented utterances. These findings revealed that native listeners’ figurative interpretation of ironic praise can change depending on indexical cues, with a reduction of pragmatic inferences in the case of foreign accent.

## Introduction

In today’s modern and globalized society, intercultural communication is largely widespread. Conversational situations where native listeners interact with foreign speakers have become increasingly common. Given this context, it becomes important to understand how language comprehension in native listeners is affected when utterances are produced by foreign speakers. Previous literature on the topic mainly focused on the impact of speaker accent on the comprehension of literal meaning. The present study explores, for the first time, the impact of a foreign accent on figurative language interpretation; a phenomenon that is pervasive in daily conversations [1,2].

Previous research seems to suggest that speaker identity can impact the way people comprehend utterances. Information that can be inferred based on the speaker’s voice (e.g., age, social status, and provenience) may have an influence on some aspects of listeners’ language comprehension [3–6]. Speaker-related effects have been reported on both lexico-semantic and syntactic analysis, with modulations of word semantic integration [7,8], and attenuated consequences of grammatical violations [9,10].

Such experimental evidence provided support for interactive models of language comprehension, claiming that different sources of information can be quickly integrated and can influence sentence analysis from the first stages of processing [11–13]. According to this perspective, world knowledge associated with speaker identity would be rapidly taken into account in order to derive the final communicative message.

The experimental evidence available so far on the role of speaker identity focuses on the way native listeners construct the literal meaning of a sentence. However, most daily conversational exchanges contain some form of figurative language [1,2], where the ultimate meaning of the discourse does not correspond to the simple sum of the lexical items. To our best knowledge, there is no study on the role of speaker identity on figurative language interpretation. The present behavioral study will fill this gap by examining the impact of speaker accent (an indexical cue to speaker identity) on figurative language comprehension, taking into account the case of irony. Offline measures of irony interpretation will be collected.

Irony is a trope that creates a contrast between a referent context (e.g., a friend has just burnt his meal) and the literal meaning of the ironic statement (e.g., “You’re such a great chef!”; [14]). The figurative meaning of irony typically corresponds to the opposite of what has been literally said [14]. In order to trigger irony interpretation, a strong expectation has to be built, and subsequently violated by the literal meaning of the statement ([14–16] note that, as the present study only collected off-line measures, it will not be possible to tease apart distinct theoretical models on the real time-processing of irony interpretation; [17,18]). Given this apparent expectation conflict, comprehenders will try to construe the meaning of the statement, making an inference about what the speaker intends to convey. Whether and what type of inferences will be made depend on a number of factors which have to do with the social norms of the communicative setting (e.g., interlocutors are supposed to exchange information that is as true, informative, relevant and clear as possible; [14]), as well as linguistic cues (e.g., prosody and intonation, [19–21]). For instance, the lexico-semantic information available in the previous discourse can have an impact on the way people attribute a figurative meaning to ironic sentences [22]. Negative contexts usually elicit stronger ironic judgements as compared to more neutral contexts (e.g., the utterance “You’re such a great chef!” is considered more ironic after a negative context–the meal is completely burnt–than after a neutral context–the meal is bland; [21,23,24]). In addition, the interpersonal relation between interlocutors seems to be another important influential factor. Shared experiences, beliefs and values among interlocutors (i.e., common ground) facilitate recognition of the final intended meaning [25]. Irony detection is impaired when listeners lack knowledge about the speaker’s linguistic and cultural background ([19]; see also [26]) and the likelihood of figurative inferences increases with the size of the common ground [27,28]. Also, what comprehenders know about the speaker’s characteristics seems to affect irony interpretation. A previous electrophysiological study showed that the same ironic statements elicited different neural responses when produced by speakers that commonly use irony as compared to ‘non-ironic’ speakers [29]. These results suggest an impact of speaker’s communicative style on online irony processing.

What is still unclear is whether listeners would reach different ironic interpretations based on speaker identity. Few behavioral studies suggest that people make different pragmatic inferences based on their expectations about the speaker. Individuals are less likely to make inferences from an under-informative statement (e.g., “some apples”) if they believe, based on previous linguistic context, that the speaker lacks knowledge of the issue of interest[30]. Importantly, listeners are less likely to make a pragmatic inference if the under-informative utterance is produced with a foreign accent, suggesting that people adjust their pragmatic interpretation depending on information inferred from a speaker’s voice [31]. Here, we explored whether a speaker’s accent modulates the perceived level of irony.

According to interactive models, world knowledge associated with speaker accent should influence the interpretation of ironic utterances [11–13]. In the case of foreign speakers, common ground should be reduced and pragmatic inferences should be less likely [19,25,27,28]. As a consequence, the final level of perceived irony should be reduced as well. Also, as irony represents a sophisticated communicative tool, listeners might not expect foreign speakers to use it (at least not as much as native speakers) presumably as a result of less refined pragmatic skills [30,31]. Thus, our main hypothesis was that speaker accent should have an impact on whether and how listeners compute pragmatic inferences ([31]; see also [15,16,25]). Native listeners should be less likely to derive figurative meanings from ironic utterances produced by foreign speakers as compared to native speakers.

### The present study

Spanish native listeners were presented with ironic and literal Spanish statements, uttered in a foreign or native accent. Participants were asked to rate the degree of irony of each story. Two different types of irony (and relative literal controls) were created: ironic criticism, where the irony was used to make an indirect critique (as in the example above “You’re such a great chef!”), and ironic praise, where the ironic statement was an indirect compliment to the interlocutor (e.g., “You’re such a horrible chef!” to a friend who has just been awarded a third Michelin star). These two types of irony have different frequency of use, with the ironic criticism being the prototypical, the most widespread, and the easiest to be learned, produced and understood [27,32,33].

For each story, accent strength and intelligibility were also measured in order to ensure that foreign accent was stronger than native accent, while keeping intelligibility constant (similarly to [9,10]).We predicted that the level of perceived irony would differ across accents, with lower scores for foreign as compared to native accented ironic statements [19,25,27,28]. This might be particularly true in the case of non-prototypical irony (ironic praise), since foreign speakers might not be expected to master such a low-frequency trope in their non-native language [30,31,34].

## Methods

### Participants

Forty-one native Spanish listeners participated in the experiment (26 women, mean age: 23, SD: 2). None of the participants reported a history of neurological, psychiatric disorders or hearing problems. All participants signed an informed consent form before taking part to the study that was approved by the Basque Center on Cognition, Brain and Language ethics committee. They received a payment of 8 € per hour.

### Materials

One hundred-twenty Spanish stories (containing 6 sentences each) were created. Each story contained three parts: a pre-target context (first four sentences), a target sentence (second-to-last sentence) and a final sentence (similarly to [35]). The context and the target sentences were manipulated so that each story had four different versions: Ironic Criticism, Ironic Praise, Literal Criticism, Literal Praise (see Table 1). Note that these four combinations were derived by a fully crossed design so that the same context could be followed by an ironic or a literal sentence and the same target sentence could have a literal or a non-literal interpretation. Between the Praise and the Criticism versions of each story, some words of the context were changed in order to make the description either positive or negative. The literal or ironic interpretation was always defined by the last word of each target sentence, which could be either positive or negative. Negative and positive target words were matched for frequency, number of letters, number of syllables, number of phonological neighbors, uniqueness point, and familiarity (all *t*s<2; EsPal;[36]).

**Table 1:** Description, examples and translations of experimental materials. L: literal; I: Ironic.

	Praise		Criticism
**L**	The context described positive events. The target sentence represents a literal compliment.		The context describes negative or undesirable events. The target sentence is a literal critical comment.
**I**	The context describes positive events. The target sentence is a compliment and requires a non-literal interpretation.		The context describes negative or undesirable events. The target sentence is a critical comment and requires a non-literal interpretation.
Spanish stories			
**L**	Mi hermano invitó a mi prima a cenar.		Mi hermano invitó a mi prima a cenar.
	Él había preparado toda la comida.		Él había preparado toda la comida.
	Mi prima me contó que estaba deliciosa.		Mi prima me contó que estaba incomestible.
	Enseguida le dije:		Enseguida le dije:
	*Es sabido que cocina muy* *bien*.		*Es sabido que cocina muy* *mal*.
	Lo pasaron muy bien charlando y riéndose.		Lo pasaron muy bien charlando y riéndose.
**I**	Mi hermano invitó a mi prima a cenar.		Mi hermano invitó a mi prima a cenar.
	Él había preparado toda la comida.		Él había preparado toda la comida.
	Mi prima me contó que estaba deliciosa.		Mi prima me contó que estaba incomestible.
	Enseguida le dije:		Enseguida le dije:
	*Es sabido que cocina muy* *mal*.		*Es sabido que cocina muy* *bien*.
	Lo pasaron muy bien charlando y riéndose.		Lo pasaron muy bien charlando y riéndose.
English translation			
**L**	My brother invited my cousin for dinner.	My brother invited my cousin for dinner.	
	He had cooked the entire meal.	He had cooked the entire meal.	
	My cousin told me that it was delicious.	My cousin told me that it was inedible.	
	Then I said:	Then I said:	
	*It is known that he cooks very* *well*.	*It is known that he cooks very* *badly*.	
	They had fun chatting and laughing.	They had fun chatting and laughing.	
**I**	My brother invited my cousin for dinner.	My brother invited my cousin for dinner.	
	He had cooked the entire meal.	He had cooked the entire meal.	
	My cousin told me that it was delicious.	My cousin told me that it was inedible.	
	Then I said:	Then I said:	
	*It is known that he cooks very* *badly*.	*It is known that he cooks very* *well*.	
	They had fun chatting and laughing.	They had fun chatting and laughing.	

Each version of the Spanish stories was auditorily presented. The stories were recorded in a Spanish native accent and British English accent (i.e., our native and foreign accent, respectively; 960 stories in total).Three native, female Spanish speakers and three native, female British English speakers recorded the sentences (each speaker recorded one third of the overall number of sentences). The native speakers were born and lived in Spain. Potential foreign speakers were selected among British people living in Spain and using Spanish on a daily basis. A Spanish native speaker qualitatively evaluated their spontaneous Spanish production in order to select speakers with a clear foreign accent and highly intelligible speech. The three British English speakers finally selected had lived and worked in Spain for at least 1 year. Participants were asked, at the end of the study, to rate the quality of their Spanish production on a scale from 1 to 5. The average was 3.8, SD: 0.7 (Speaker 1: 4.0, SD 0.6; Speaker 2: 3.7, SD 0.7; Speaker 3: 3.6, SD 0.7).

During the recording, all speakers were asked to maintain neutral intonation and a slow speech rate (see Table 2 for the acoustic features of each condition). To minimize prosodic differences across conditions, target sentences were recorded separately from the contexts and later edited in order to be preceded by different contexts. To minimize possible differences in speech rate across accents, each native speaker was asked to listen to the corresponding foreign speakers’ stories (as in [4,5,10]). This procedure might have reduced the naturalness of the recorded sentences, but it allowed us to control for potential confounds, such as speech rate and duration, which can distinctively characterize ironic and literal utterances and influence pragmatic judgements [37,38]. Story recordings were normalized with respect to average root mean squared amplitude.

**Table 2:** Acoustic features of the stimuli divided by experimental conditions. Values reported for Ironic Criticism and Literal Praise were identical since those conditions were built using the same target sentence (the same was true for Ironic Praise and Literal Criticism). SD are reported in parenthesis. Values of duration and pitch are reported for both the target sentence and the target word.

	Durations (ms)		Speech rates (words/sec)	Pitch Mean (Hz)		Pitch Span (Hz)	
	Sentence	Word	Sentence	Sentence	Word	Sentence	Word
**Native accent**							
Ironic Criticism- Literal Praise	1992 (459)	657 (153)	.39 (.09)	177 (15)	159 (16)	126–263	132–217
Ironic Praise- Literal Criticism	1986 (463)	645 (184)	.39 (.09)	177 (14)	159 (16)	123–258	132–209
**Foreign Accent**							
Ironic Criticism- Literal Praise	1901 (481)	619 (149)	.37 (.09)	212 (10)	183 (18)	135–301	142–233
Ironic Praise- Literal Criticism	1919 (500)	620 (182)	.38 (.09)	212 (11)	183 (23)	129–307	138–239

Since speech rate and pitch were maintained constant across story types (see Table 3), the distinction between ironic criticism and ironic praise was mainly based on lexical cues (rather than prosodic cues). Overall, recordings did not differ in duration, speech rate and pitch across story types and accents. The only difference was that foreign speakers showed slightly higher pitch as compared to native speakers. Importantly, differences in speech rate, pitch, and duration could not explain any significant interaction in the final irony ratings since all interactions involving these acoustic features were not significant (all *F*s<1.2; all *p*s>0.20; see Table 3). Eight experimental lists of 120 trials (15 items per condition) were created so that each version of the stories appeared only once per list. Half of the trials were uttered in a native accent and the other half in a foreign accent.

**Table 3:** Statistical results relative to the acoustic features of the experimental stimuli. No interaction between Story Type (two levels: Ironic Criticism- Literal Praise; Ironic Praise-Literal Criticism) and Accent (two levels: Foreign; Native) was significant.

	Durations				Speech Rate		Pitch Mean			
	Sentence		Word		Sentence		Sentence		Word	
	*F*	*p*	*F*	*p*	*F*	*p*	*F*	*p*	*F*	*p*
**Accent**	1.75	.19	2.80	.10	2.31	.13	486.77	< .001	112.34	< .001
**Story Type**	.19	.66	.29	.59	.47	.49	.25	.62	.002	.96
**Accent x Story Type**	.76	.39	.41	.52	1.14	.29	.13	.72	.000	.99

### Procedure

Participants were seated in a sound-attenuated room in front of a computer screen and were asked to complete an online survey. They were presented with one auditory story at a time and were required to rate it based on three different dimensions: accent strength, intelligibility and irony. Accent strength was measured on a scale from one to five, where one was a light accent and five, a strong accent. For intelligibility, participants were asked to transcribe the second-to-last word of each story. Irony was rated on a scale from one to five, where one was literal and five was ironic. To make sure participants understood the task and used the rating scale of irony in the same way, the instructions provided examples of ironic and literal stories, which were representative of the experimental material (the distinction between sarcasm and ironic criticism might not be straightforward, and potential overlaps are possible- see [27] for further discussion. It is beyond the aim of this study to distinguish between these two categories). At the end of the survey, participants had to classify each of the six speakers as a native or a foreign speaker and guess her mother tongue. After providing their opinion about the speaker’s mother tongue, participants had to rate their familiarity with English accented Spanish (no distinction between different types of English accent was made) and provide their opinions about the frequency of irony usage in different countries. The experimental session lasted about an hour and half.

### Statistical analyses

The analyses were carried out fitting linear mixed-effect models to our data [39] with Accent (Native, Foreign), Polarity (Criticism, Praise), and Story (Literal, Ironic) as predictors. The reference levels chosen to estimate the intercept were Native, Criticism, and Literal. Analyses were carried out using the *lme4* package [40]. The p-values of the linear mixed-effect models were calculated treating the *t* statistic as if it were a *z* statistic.

Model comparisons were performed using likelihood ratio tests and a forward-testing approach. A series of models was built with increasing complexity in the random-effects structure [41] for each of the three dependent variables (Accent Strength, Intelligibility, Irony). The null model included the three fixed effects (Accent, Polarity, Story) and the by-subject random intercept. Subsequently, more complex models were built including the by-item random intercept, the fixed effects interactions, and the by-subject random slopes. These factors were included one at a time and their contribution to improving model fit was evaluated by comparing the model with a similar one that did not include the factors in question. Our model selection heuristic tested for random slopes inclusion/exclusion, leaving in by default the by-subject and by-item random intercepts (as in [41]). We attempted to reach the maximal random-effects structure, but the model was simplified and more complex models were disregarded when the *p*-value for the significance of the difference was above 0.20 [42]. Model comparisons carried out to identify the best-fit model are included in supplementary material (S1 File). To test differences between the factor levels that could not be read directly from the model, post hoc tests were performed using *lsmeans* package [43]. FDR correction for multiple comparisons was applied on follow-up tests. Effect sizes were defined as the fixed effect coefficient over its SE [44]. Data and scripts are available on Open Science Framework (https://osf.io/m8ynw/).

## Results

At an assessment with a native speaker participants’ English production was rated from 1 to 5 (1: total lack of knowledge; 5: extremely high proficiency level). Participants had an intermediate to high knowledge of English (range: 3–5; average: 3.65, SD: 0.62). All participants were familiar with English accented Spanish and they reported being exposed to English accented Spanish on a daily basis (average of hours per week: 3, SD 5; average of English-Spanish speakers participants were in contact with: 3, SD: 5). They correctly recognized which speakers had a foreign accent and which speakers had a native accent. Moreover, they correctly recognized the mother tongue of the native speakers, and 70% of the participants correctly recognized the mother tongue of the foreign speakers. When they were explicitly asked to rate the use of irony in Spain and English-speaking countries (United Kingdom, USA) on a scale from one to five (one: they use irony very little; five: they use irony a lot), the score of English irony (i.e., amount of irony used in English speaking countries, 3.57, SD: 0.75) was lower as compared to Spanish irony (4.59, SD: 0.63; *t* = 5.96, *p* < .001).

### Accent strength

**Fig 1:**
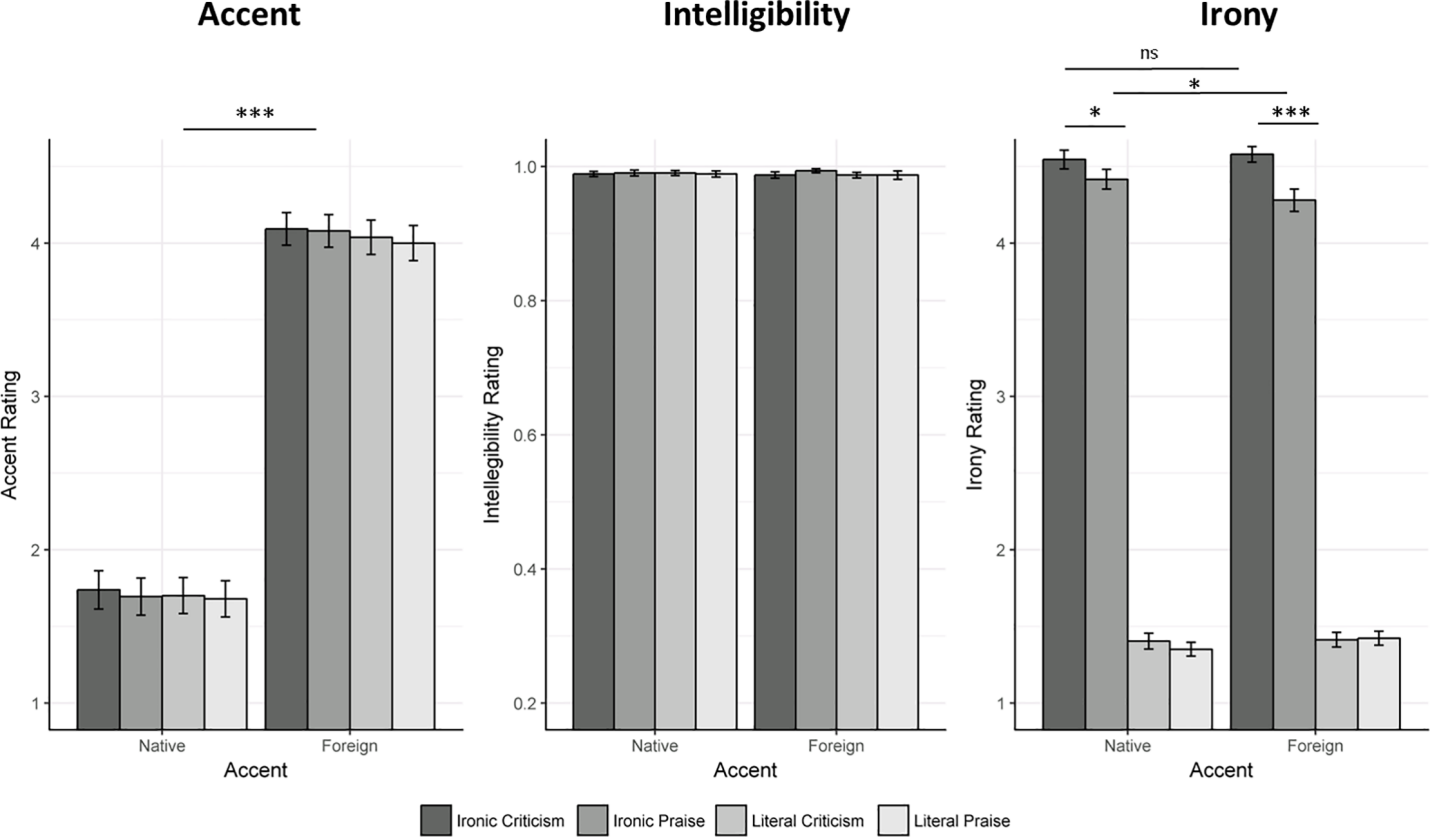
Bar plots of accent, Intelligibility and Irony average scores. Standard error bars are displayed for each experimental condition.

The best-fit model included the three fixed effects, the by-subject and by-item intercepts, and the by-subject random slope of the Accent factor (*X*^2^(3) = 2229, *p* < .001). The stories uttered with a foreign accent were rated as more accented as compared to the stories uttered with native accent (intercept: 1.70, SE: 0.12; β = 2.35, SE: 0.18, *t* = 13.08, *p* < .001; *D* = 13.06). No other fixed effect was significant (Fig 1). Adding the fixed effects interactions did not lead to improvements of the model fit (*X*^2^<1; see S1 File).

### Intelligibility

The best-fit model included the three fixed effects and the by-subject and by-item intercepts (comparison with the null model: *X*^2^(1) = 39.88, *p* < .001). No fixed effect was significant (intercept: 0.99, SE: .004, all *t*s<1; Fig 1). Each experimental condition showed an average intelligibility score above 97%, indicating that participants could easily identify words regardless of story type, polarity and accent. Adding the fixed effects interactions did not lead to improvements of the model fit (*X*^2^<1.5; see S1 File).

### Irony

The best-fit model included the three fixed effects, the by-subject and by-item intercepts, the interaction Accent x Polarity x Story, and the random slope of Story and Polarity factors (final nested comparison: *X*^2^(3) = 6.01, *p <* .20; see S1 File). Overall, the ironic stories were considered more ironic than the literal stories (intercept: 1.46, SE: 0.03; β = 3.06, SE: 0.03, *t* = 116, *p* < .001, *D* = 102). Adding the two-way interaction Story x Polarity and the three-way interaction Accent x Story x Polarity to simpler models significantly improved the model fit (*X*^2^(1) = 13.44, *p <* .001; *X*^2^(2) = 5.96, *p <* .05). Post hoc tests corrected for FDR revealed that there was no significant difference between literal praise and literal criticism, which was true for each accent (native: β = 0.05, SE: 0.05, *t*<1, *p* = .40, *D* = 1; foreign: β = -0.01, SE: 0.05, *t*<1, *p* = .86, *D* = 0.2; Fig 1). Also, each literal version of the story was similarly rated across accents (literal praise: β = -0.07, SE: 0.05, *t* = 1.40, *p* = .20, *D* = 1.4; literal criticism: β = -0.01, SE: 0.05, *t*<1, *p* = .86, *D* = 0.2). There was a significant difference between ironic praise and ironic criticism, with the former rated less ironic that the latter (native: β = 0.13, SE: 0.05, *t =* 2.37, *p* < .05, *D* = 2.6; foreign: β = 0.30, SE: 0.05, *t* = 5.52, *p <* .001, *D* = 6). This was true for both accents, but the difference was greater in the case of the foreign accent. While the score for ironic criticism was similar across accents (β = -0.03, SE: 0.05, *t*<1, *p =* .56, *D* = 0.6), ironic praise stories were considered less ironic in the case of foreign accent than in native accent (β = 0.14, SE: 0.05, *t* = 2.68, *p <* .05, *D* = 2.8).

Finally, we explored the relation between participants’ English proficiency scores and irony ratings of the foreign accent conditions. No significant correlation was found (all |*r*s| < .10, all *p*s>.50).

## Discussion

The present study examined the impact of speaker accent on irony interpretation. Native listeners were presented with Spanish auditory stories in native and foreign accents. Four types of stories were presented: ironic criticism, ironic praise, literal criticism, and literal praise. Foreign and native accented stories differed in accent strength and had similarly high levels of intelligibility, confirming the quality of our experimental material. No differences across story types were found for accent strength and intelligibility, meaning that these factors could not contribute to explaining different levels of irony across stories.

The results of the irony ratings revealed that literal stories (literal praise and criticism) were rated similarly across accents, showing that the main comprehension and access to meaning of the stories with a literal interpretation were not drastically affected by speakers’ accents. Interestingly, despite this overall similarity in interpreting accented and unaccented stories, the results for irony scores showed a clear effect of speaker accent. While ironic criticism was similarly rated in both accents, ironic praise was judged less ironic when produced by foreign speakers as compared to native speakers. In addition, ironic praise always showed a lower irony score as compared to ironic criticism (in line with previous findings, [23,24]; see [20] for potential explanations), but this difference was greater in the foreign accent. When ironic praise was produced by foreigners (relative to natives) listeners were more likely to interpret sentences as literal, to report uncertainties and low levels of irony (see S2 File).

The present findings showed, for the first time, an impact of the speaker’s voice characteristics on the interpretation of a certain type of irony. Information conveyed by the speakers’ accent (e.g., being native or foreign) can be actively used by the listeners and change the final interpretation of ironic praise.

These findings are in line with previous research [4,5,7,9,10], showing an impact of speaker accent on language comprehension. The present results are also in line with interactive models of language comprehension [11–13] and inform models of figurative language analysis [14,23], showing that not only the referent context but also the speaker accent can influence irony interpretation. The results are compatible with the idea that listeners are less likely to produce pragmatic inferences when common ground is reduced[19,25,27,28]. However, the notion of common ground cannot explain why the reduction of perceived irony was observed only in a certain type of irony (ironic praise).

The fact that only low-frequency ironic statements were considered less ironic when pronounced by foreign speakers might be the result of listener’s specific expectations triggered by the speakers’ accent [16]. Here below we mention a series of possible accounts. One possibility is that that listeners assume foreign speakers to be less pragmatically competent than native speakers [30,31,34], or at least not competent enough to be able to use low-frequency tropes. Listeners might know that foreign speakers do not always reliably convey their intended meanings and do not typically use complex rhetoric constructions. As a consequence, native listeners might hesitate to infer figurative meaning from infrequent ironic statements. Another possibility, partially related to the previous one, is that knowing how difficult it is to learn a second language, listeners would be more likely to consider multiple alternative interpretations especially in case of potential ambiguity, such as ironic praise [15]. One last possibility is that native listeners activate specific stereotypical information associated with speaker identity, which drives their inferences about speakers’ intended meaning [8,13]. In the present study, most participants identified foreign speakers to be English native speakers and they also estimated the frequency of use of irony as being lower in English-speaking countries as compared to Spain. This stereotypical knowledge about the usage of irony could have shaped listeners’ non-literal interpretation of irony. Note that in the present study the stereotypical knowledge related to usage of irony was measured without distinguishing between praise and criticism. Thus, it is not possible to know whether this type of knowledge played a role in listeners’ interpretation. Future research is needed in order to better define native listeners’ expectations regarding figurative language uttered by foreign speakers.

Note that the present results might not exclusively concern foreign accents. Following the potential explanations mentioned above, similar findings might be observed any time the speaker’s voice triggers specific expectations about the interlocutor’s linguistic background, as it might be the case of heritage speakers and low-proficient native speakers. Further studies are needed in order to confirm this more general hypothesis.

Although the present study showed an impact of speaker accent on ironic praise, it is also worth noting that other experimental conditions did not seem to be strongly affected by accent, such as literal stories and ironic criticism. The null result of accent in the case of literal language seems to be at odds with previous studies showing a modulation of language comprehension processes as a function of accent [3–10]. One potential explanation of this inconsistency might be due to the type of technique used. While previous accent effects on literal language have been reported using online measures (either ERPs or fMRI correlates), the present study collected behavioral measures, which cannot inform on different stages of irony processing. Finally, the null effect of accent for ironic criticism might suggest that accent selectively impacts some types of figurative language and not others. This distinction might have to do with the frequency, the valence or the conventionality of these tropes [20]. However, we think we need to be careful when interpreting both null results for at least two reasons. First, a null effect does not provide evidence for the lack of the effect. Second, we cannot exclude that our design might not be sensitive enough to these manipulations as we only collected offline explicit measures. More implicit behavioral measures (which are less influenced by strategic behaviors) together with electrophysiological responses (which can monitor online processes) are needed to further confirm and complement these findings.

To conclude, the present study explored the impact of speaker accent on figurative language comprehension (irony). The results showed that native listeners adjust their figurative interpretation of ironic praise based on indexical cues.

## Supporting information

S1 File Model comparisons. For each variable taken into account, a table showing the model comparisons is displayed.
(DOCX)

S2 File Density plots. The conditions of foreign and native accented ironic praise are examined closely through a density plot.
(DOCX)

S3 File Raw data from the survey. Each participant and item score is reported for Accent Strength, Intelligibility and Irony.
(XLSX)
