# Supplementary material for: The impact of foreign accent on irony interpretation
Source: PLoS One. 2018 Aug 8;13(8):e0200939. doi: 10.1371/journal.pone.0200939 (PMC6082519; doi:10.1371/journal.pone.0200939)
Supplement: S1 File — For each variable taken into account, a table showing the model comparisons is displayed. (DOCX) [file pone.0200939.s001.docx]

Model comparisons for Accent Strength

Better-fit models were defined any time the p-value for the significance of the difference was below 0.20 (Matuschek et al., 2017). The best-fit model corresponded to the last model that improved the model fit. The maximal converging model was the model that did converge and included the maximal random-effects structure.

| **Model** | **Comparison** | **Fixed effects** | **Random effects** | **df** | **χ^2^** | ***p*** |
| --- | --- | --- | --- | --- | --- | --- |
| Null |  | Accent + Story + Polarity | (1 \| subject) |  |  |  |
| 1 | Null |  | (1 \| item) | 1 | 0.41 | 0.52 |
| 2 | 1 | Accent x Story |  | 1 | 0.64 | 0.42 |
| 3 | 1 | Accent x Polarity |  | 1 | 0.02 | 0.89 |
| 4 | 1 | Story x Polarity |  | 1 | 0.0002 | 0.99 |
| 5 | 1 | Accent x Story x Polarity |  | 4 | 0.87 | 0.93 |
| 6* | 1 |  | (1 + Accent \| Subject) | 3 | 2229.3 | <0.001 |
| 7 | 6 |  | (1 + Story \| Subject) | 3 | 1.13 | 0.77 |
| 8^ | 6 |  | (1 + Polarity \| Subject) | 3 | 1.28 | 0.73 |

^Maximal converging model: Accent + Story + Polarity + (1 | subject) + (1 | item) + (1 + Accent | Subject) + (1 + Story | Subject)+ (1 + Polarity | Subject)

*Best-fit model: Accent + Story + Polarity + (1 | subject) + (1 | item) + (1 + Accent | Subject)

Model comparisons for Intelligibility

| **Model** | **Comparison** | **Fixed effects** | **Random effects** | **df** | **χ^2^** | ***p*** |
| --- | --- | --- | --- | --- | --- | --- |
| Null |  | Accent + Story + Polarity | (1 \| subject) |  |  |  |
| 1* | Null |  | (1 \| item) | 1 | 39.88 | <0.001 |
| 2 | 1 | Accent x Story |  | 1 | 0.33 | 0.56 |
| 3 | 1 | Accent x Polarity |  | 1 | 0.32 | 0.57 |
| 4 | 1 | Story x Polarity |  | 1 | 0.69 | 0.41 |
| 5 | 1 | Accent*Story x Polarity |  | 4 | 1.44 | 0.84 |
| 6^ | 1 |  | (1 + Accent \| Subject) | 3 | 0.35 | 0.95 |
| 7 |  |  | (1 + Story \| Subject) | model did not converge | | |
| 8 |  |  | (1 + Polarity \| Subject) | model did not converge | | |

^Maximal converging model: Accent + Story + Polarity + (1 | subject) + (1 | item) + (1 + Accent | Subject)

*Best-fit model: Accent + Story + Polarity + (1 | subject) + (1 | item)

Model comparisons for Irony

| **Model** | **Comparison** | **Fixed effects** | **Random effects** | **df** | **χ^2^** | ***p*** |
| --- | --- | --- | --- | --- | --- | --- |
| Null |  | Accent + Story + Polarity | (1 \| subject) |  |  |  |
| 1 | Null |  | (1 \| item) | 1 | 1.66 | 0.20 |
| 2 | 1 | Accent x Story |  | 1 | 3.05 | 0.08 |
| 3 | 2 | Accent x Polarity |  | 1 | 1.07 | 0.30 |
| 4 | 2 | Story x Polarity |  | 1 | 13.44 | <0.001 |
| 5 | 4 | Accent x Story x Polarity |  | 2 | 5.96 | <0.05 |
| 6 | 5 |  | (1 + Accent \| Subject) | 3 | 0.10 | 0.99 |
| 7 | 5 |  | (1 + Story \| Subject) | 3 | 207.39 | <.001 |
| 8*^ | 7 |  | (1 + Polarity \| Subject) | 3 | 6.01 | 0.11 |

^Maximal converging model: Accent + Story + Polarity + Accent x Story x Polarity + (1 | subject) + (1 | item) + (1 + Accent | Subject) + (1 + Story | Subject) + (1 + Polarity | Subject)

* Best-fit model coincides: Accent + Story + Polarity + Accent x Story x Polarity + (1 | subject) + (1 | item) + (1 + Story | Subject) + (1 + Polarity | Subject)
