# Supplementary material for: The impact of foreign accent on irony interpretation
Source: PLoS One. 2018 Aug 8;13(8):e0200939. doi: 10.1371/journal.pone.0200939 (PMC6082519; doi:10.1371/journal.pone.0200939)
Supplement: S2 File — The conditions of foreign and native accented ironic praise are examined closely through a density plot. (DOCX) [file pone.0200939.s002.docx]

*
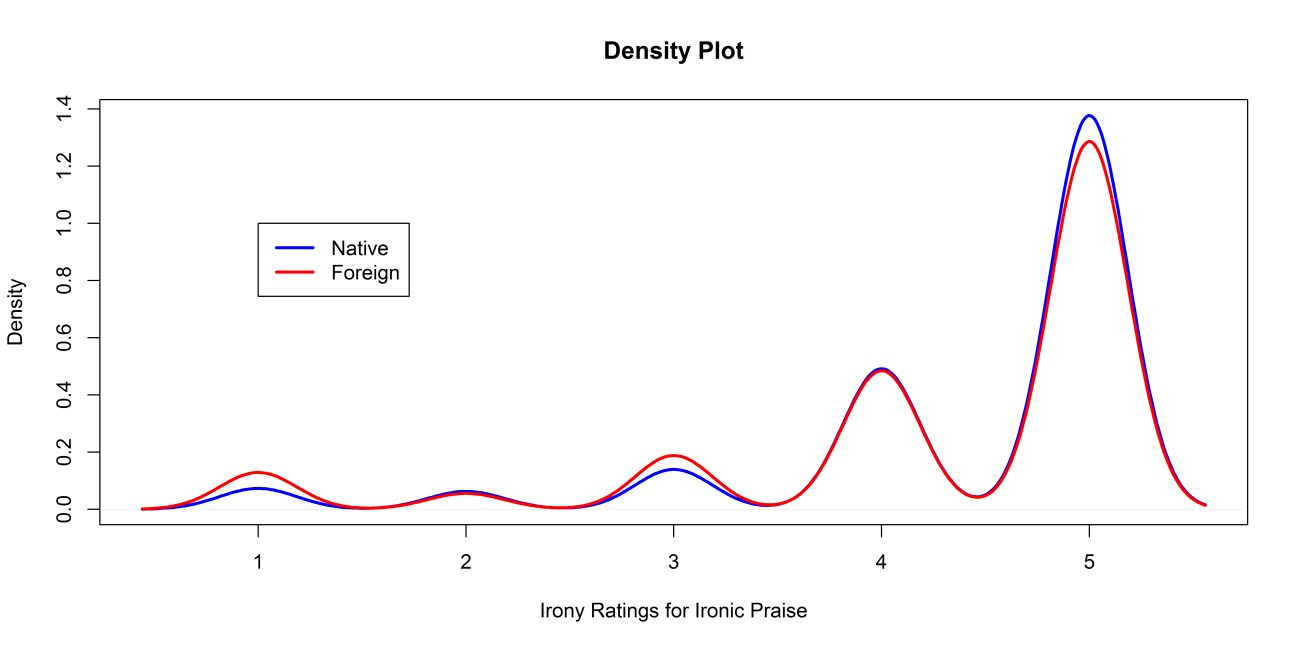
*

The decreased degree of irony observed in the case of foreign accented irony praise can be due to two different reasons:

1. native listeners less often understand the ironic intent of the foreign speakers as compared to the native speakers and they treat ironic stories as literal;
2. native listeners consider foreign accented ironic praise less ironic than native accented ones and they rate them with a lower degree of irony.

The density plot suggests that our data present a mixture of these two possibilities. For instance, having a foreign accent makes literal interpretations more frequent (i.e., for score 1, there is higher density in foreign accent than in native accent), but also increases uncertainty (i.e., for score 3, there is higher density in foreign accent than in native accent) and reduced the frequency of reporting the highest level of irony (i.e., for score 5, there is lower density in foreign accent than in native accent).
